# Investigating the Conformation of S100β Protein Under Physiological Parameters Using Computational Modeling: A Clue for Rational Drug Design

**DOI:** 10.2174/1874120701812010036

**Published:** 2018-06-29

**Authors:** Elvis K. Tiburu, Ibrahim Issah, Mabel Darko, Robert E. Armah-Sekum, Stephen O. A. Gyampo, Nadia K. Amoateng, Samuel K. Kwofie, Gordon Awandare

**Affiliations:** 1 Department of Biomedical Engineering, College of Basic and Applied Sciences, University of Ghana, P. O. Box LG 25, Legon, Ghana; 2 Department of Biochemistry, Cell and Molecular Biology, University of Ghana, P. O. Box LG 25, Legon, Ghana; 3 West African Centre for Cell Biology of Infectious Pathogens, University of Ghana, P. O. Box LG 25, Legon, Ghana

**Keywords:** S100β Protein, Molecular Dynamics, Cofactors, Energy Minimization, Physiological Parameters, Alzheimer's

## Abstract

**Background:**

Physiochemical factors such as temperature, pH and cofactors are well known parameters that confer conformational changes in a protein structure. With S100β protein being a metal binding brain-specific receptor for both extracellular and intracellular functions, a change in conformation due to the above-mentioned factors, can compromise their cellular functions and therefore result in several pathological conditions such as Alzheimer’s disease, Ischemic stroke, as well as Myocardial Infarction.

**Objective:**

The studies conducted sought to elucidate the effect of these physiological factors on the conformational dynamics of S100β protein using computational modeling approaches.

**Method:**

Temperature-dependent and protein-cofactor complexes molecular dynamics simulations were conducted by varying the temperature from 100 to 400K using GROMACS 5.0.3. Additionally, the conformational dynamics of the protein was studied by varying the pH at 5.0, 7.4 and 9.0 using Ambertools17. This was done by preparing the protein molecule, solvating and minimizing its energy level as well as heating it to the required temperature, equilibrating and simulating under desired conditions (NVT and NPT ensembles).

**Results:**

The results show that the protein misfolds as a function of increasing temperature with alpha helical content at 100K and 400K being 57.8% and 43.3%, respectively. However, the binding sites of the protein was not appreciably affected by temperature variations. The protein displayed high conformational instability in acidic medium (pH ~5.0). The binding sites of Ca^2+^, Mg^2+^ and Zn^2+^ were identified and each exhibited different groupings of the secondary structural elements (binding motifs). The secondary structure analysis revealed different conformational changes with the characteristic appearance of two beta hairpins in the presence of Zn^2+^and Mg^2+^.

**Conclusion:**

High temperatures, different cofactors and acidic pH confer conformational changes to the S100β structure and these results may inform the design of novel drugs against the protein.

## 1 INTRODUCTION

### 1.1 Background

**Fig. (1):**
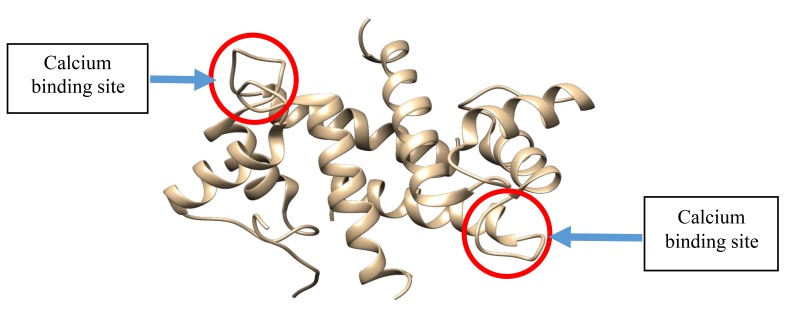
A PyMol representation of the native structure of S100β protein (PDB ID: 5D7F), depicting the two calciumbinding sites circled in red colour.

S100β protein is a metal-binding receptor located in the metal ion-rich synaptic regions of the human brain. At the onset of any neurodegenerative disorders such as Alzheimer’s, Down’s syndrome, trauma, brain injury, myocardial infarction and stroke, there is an increase in the concentration of S100β proteins which serves as a biomarker for clinical purposes [1]. The presence of metal ions in physiological environment makes metal-binding a necessary proviso for triggering conformational changes which ultimately can result in biological activity [2, 3]. Conformational changes in proteins refer to an alteration in the tertiary structure of the protein influenced by physiochemical conditions such as temperature, pH and ionic binding [4, 5]. The failure of S100β protein to maintain its native functional three-dimensional (3D) conformation has been linked to pathological conditions known as neurodegenerative disorders [6] (Fig. **1**). Although native S100β has two helix-loop-helix calcium binding structural motifs, under unfavourable physiochemical conditions (*i.e.* temperature, pH and cofactors), these proteins experience a change in their native 3D structure [7].

The protein is a member of the family of calcium-binding proteins known as the S100 family [8, 9]. The biological function of these proteins is regulated when bound to calcium ions. The family consists of 25 members which are functionally categorized into three groups: those that perform intracellular regulatory effects, extracellular regulatory effects as well as the combination of the two [10]. The members of this family are considered to be low molecular weight proteins with an average monomer molecular weight of around 12-14 kDa but 21-23 kDa in its homodimer structure. They are specifically found in vertebrates, suggesting that they are evolutionarily young [11].

S100β proteins are basically characterized by two EF-hand (pseudo and typical EF-hand) calcium active binding motifs and so undergoes a large conformational change upon binding with calcium. However, it has been shown that the pseudo-EF-hand has minor structural changes when Ca^2^+ binds whereas the typical has a large repositioning of several side chain oxygen ligand during Ca^2+^ coordination (EF-2: D61-E72). Several target structures have been exploited including Zn^2+^ site in S100β inhibition which is relevant in drug design, hence can mimic target binding which can enhance Ca^2+^ binding affinity. This exposes the hydrophobic residues and results in protein-protein interaction affecting biological activities in the cellular environment.

A typical dimeric S100-target interaction is Ca^2+^- dependent and involves at least 11 possible states, 13 dissociation constants, 4 conformational changes and the corresponding rate constants, per symmetric subunit. Furthermore, Ca^2+^ binding to S100β causes conformational changes and the binding events for a single target are symmetric and occur independently of those on the other subunit, thereby making it interesting to consider one S100β subunit in thermodynamic scheme [12].

These proteins also show a high degree of specialization by their cell-specific expression patterns. For instance, S100β is brain-specific while S100α1 is restricted to cardiomyocytes and cells of the kidney only. S100β proteins are characterized by two calcium-binding active sites and the name S100 is coined because they are soluble in 100% saturated ammonium sulphate at neutral pH [10, 11]. Exogenous S100β excite the survival of neurons together with neuritis extension which can affect learning and memory in a chemical regulatory (paracrine, autocrine, endocrine) manner [13]. S100β also plays a role as a mediator of glial-neuronal interactions to enhance brain development and synaptic transmission possibly through G-protein coupled receptor (GPCR). Furthermore, they play a role in raising intracellular calcium concentrations either by dependent *Ca^2^*^+^- channels or by depletion of the calcium stores [14]. At the molecular level, S100β proteins modulate several biological activities such as calcium homeostasis, protein phosphorylation, motility, energy metabolism, maintenance of cytoskeleton dynamics, cell growth, as well as regulation of cell proliferation, inflammation, conduction and transmission of nerve impulses [15, 16].

Temperature variations disrupt hydrogen bonds and non-polar hydrophobic interactions of a protein due to increase in its molecular kinetic energy. This may lead to denaturing of the protein. Thus, temperature variations confer unique conformational changes and alter the biological activities of the protein. Additionally, the pH dependent property through the pKa values of the protonated residues of the protein confers different conformational dynamics to the S100β protein. Much more, biological activities of proteins are mostly modulated by small molecules (cofactors or ligands). Upon ligand or cofactor binding, a receptor undergoes a structural change which triggers a specific biological response that may be critical to the functions of an organism at the cellular and molecular level [17]. For example, the process of phosphorylation activates or deactivates a receptor thereby altering its biological activity. The binding affinity between a cofactor and a protein as well as its specific residues at the binding motifs play a pivotal role in the conformational dynamics of the protein [18]. In this study, computational modeling approaches were used to predict the conformational changes of S100β protein at varying physiological conditions such as temperature, pH and cofactors. Since the structure and function of a protein are intrinsically interrelated, their comprehension is indispensable for drug design.

### 1.2 Neurodegenerative Disorders with Increased S100β Proteins Concentrations

#### 1.2.1 Ischemic Stroke

Ischemic Stroke (IS) is the leading cause of disability and death associated with old age. Health statistics revealed that with over 15 million cases of strokes recorded annually worldwide, methods to effectively diagnose and monitor therapy are needed [19]. The use of blood biomarkers is one of the methods that hold great potential in predicting the risk of stroke, diagnosing stroke as well as in monitoring treatment. Studies also reveal that among the two types of stroke (Ischemic and Haemorrhagic stroke), 87% of diagnosed stroke cases are of Ischemic nature [1, 2]. IS occurs when brain cells die due to inadequate blood flow as a result of blockage of blood vessels leading to the brain [20]. At the onset of this neural disorder, S100β proteins are released continuously from the glial and Schwann cells into the blood and Cerebrospinal Fluid (CSF). This is due to astrocytic activation caused by deprivation of oxygen and nutrients [7]. Thus, the concentration of S100β proteins increases significantly at the onset of IS and correlates with infarct volume, stroke severity and functional outcome [8]. Hence, clinically, increased concentrations of S100β proteins in the blood and CSF serve as a useful neuro-biomarker in the diagnosis and long-term prediction of clinical outcomes pertaining to IS. The stressful physiochemical conditions in brain Ischemia results in protein misfolding and aggregation of abnormal proteins [21]. Due to the fact that the native three-dimensional conformation accounts for proper cellular function of a protein; any alteration in this tertiary structure either leads to a gain of other toxic functions or loss of key functions (*e.g.* the loss of motor functions which is prevalent in stroke patients). Hence, conformational changes due to unfavourable cellular conditions such as ion, pH, temperature and ligands are important in the progression of protein misfolding pathologies as well as altering of biological activity.

#### 1.2.2 Alzheimer’s Diseases

Alzheimer's Disease (AD) is a neuropathological ailment which is characterized by a gradual increase in dementia. This kind of dementia impairs memory, thinking and behaviour. Symptoms usually progress slowly and worsen with time, becoming profound enough to inhibit the performance of daily tasks [22]. The characteristic neuro-pathological markers of AD are the formation of amyloid plaques and tangling of the neuro-fibrils. Patients suffering from AD experience a significant increase of S100β protein levels due to conformational changes as well as a seeming interplay between ß-amyloids and expression of S100β protein. The ß-amyloid stimulates the synthesis of both S100β mRNA and S100β protein in astrocyte cultures. Extracellular S100β might contribute to inflammations in the brain regions by astrocytes and microglial neurons activations. The inflammation affects the expression of S100β proteins, making it pathologically relevant in the degeneration of the Central Nervous System (CNS) in AD [13].

#### 1.2.3 Myocardial Infarction

Myocardial Infarction (MI), usually known as heart attack occurs when there is a decreased perfusion to the heart tissues as a result of blockage of the left coronary artery. This results in an increased myocardial metabolic demand causing a decreased supply of nutrients and oxygen to the myocardial cells *via *the coronary circulation [14]. S100β is a well-known protein marker of brain damage. At the onset of MI, there is an increased concentration of S100β protein in the serum. This shows that, a careful assessment of the function and the structural alterations of these proteins in heart attack progression might contribute to a better understanding of the regulatory and counter regulatory mechanisms underlying the progressive decline in cardiac function in patients with myocardial infarction [15].

### 1.3 Computational Modelling *via *Molecular Dynamics (MD) Simulations

Computational modelling is basically the use of computational techniques to simulate and study the behaviour of complex systems using mathematics, physics, informatics, control theories and computer science. This platform uses various methods to desegregate tentative and scientific data into models used to demonstrate biomedical phenomena and exploit system responses, predictions and develop hypothesis [23]. From the above stems molecular dynamics simulation that simulates the trajectory of a system of particles. This is prominent for the generations of non-equilibrium ensembles as well as dynamic events analysis. Molecular dynamics simulation is based on Newton’s equation of motion represented as:

$\left(\frac{d^{2}r_{i}}{dt^{2}}=\frac{F_{i}}{m_{i}}\right)$

where, *F_i_* is the force on any atom, *r_i_* is the positions of atoms in the system and *m_i_* is the atom mass.

This equation of molecular dynamic simulations is prominent in classical mechanics and from it stems varied algorithms to simulate trajectories of a system of particles [24]. Thus, the movement of the atoms is simulated by numerically solving the Newton’s equations of motion based on the summation of the forces between non-bonded and bonded atom interactions as well as restrains and/or external forces. This allows the determination of the potential and kinetic energies of the atoms as well as its pressure tensor [25]. These simulations help in determining the structural alterations of complex systems and are employed in studying the conformational dynamics of S100β protein at varying physiochemical parameters to unravel new knowledge in supporting clinical decisions.

## 2 MATERIALS AND METHOD

The X-ray crystal structure of the S100β protein (PDB ID: 5D7F) with a resolution of 1.3Å was acquired from Protein Data Bank (PDB) [26]. PDB files contain standard atomistic coordinates, experimental method, resolution and secondary structure of a protein [26]. Chain A of the 5D7F structure was utilized in the computational modeling since it binds to more glycerol ligands as opposed to the chain B from the solved structure. The equilibrium structure of S100β was obtained by a long-time-scale molecular dynamics (MD) simulation using GROningen MAchine for Chemical Simulations (GROMACS) 5.0.3 [27]. Chiron [28] and YASARA [29] servers were used to predict the steric clashes, void volume and optimization of the protein. Single Point Charge (SPC) water model was used to solvate the protein without considering hydration shells. Furthermore, equivalent number of Na^+^ ions were added to make the simulation system electrically neutral to determine the molecular conformation of the protein. Energy minimization was applied to reduce and stabilize the large forces and structural distortions due to the number of hydrogens added as well as the broken hydrogen bond networks. The pressure, temperature and volume equilibration of the system was performed using the total number of Particles-Pressure-Temperature (NPT) and Particles-Volume-Temperature (NVT) ensembles for 1000 ns, and the coordinates were saved at 1 ps frame intervals.

All the simulations were conducted utilizing the Optimized Potential for Liquid Simulations All Atom (OPLS AA) force fields and Assisted Model Building with Energy Refinement (AMBER) force fields [30, 31]. GROMACS 5.0.3, a platform used to perform molecular dynamic simulations and energy minimization utilities such as sasa, hbonds, rms and rmsf were used to analyse the simulation data [27]. The same simulation protocol was used to assess the conformational dynamics of the protein regarding cofactor-bound S100β protein as well as temperature variations. The pressure equilibration algorithm employed was the Berendsen coupling thermostat scheme. The long range electrostatic interactions of the protein were calculated using the Linear Constraint Solver (LINCS) algorithm defined in GROMACS with a cut-off length of 1A^o^ for the Van der Waals interactions. The trajectories were saved every 100 ps frame and the total NPT and MD run simulation time was 10000 ps for each case of temperature simulated. The resulting structures from the simulations were uploaded to Protein Data Bank Summary (PDBsum) Server for secondary structure contents analysis [32]. Ambertools17 [30] was used to protonate the amino acid residues of the protein to mimic the various pH values using the constant pH molecular dynamics (CpHMD) techniques. Metal Ion Binding (MIB) server was also utilized to assess the binding sites of the protein-cofactor complex [33]. Open Babel software [34] was also used to convert PQR file to PDB file format. Xmgrace plotting tool was used to plot the data obtained from the MD simulations. Statistical analysis was performed using IBM SPSS Statistics version 20 [35]. Statistical computations were undertaken using 95% confident intervals to determine the significant differences among the results obtained from the simulations. The error bars in the graphs also depicts statistically significant differences among the quantitative results obtained from the simulations.

## 3 RESULTS AND DISCUSSION

### 3.1 pH-Induced Conformational Changes

**Fig. (2):**
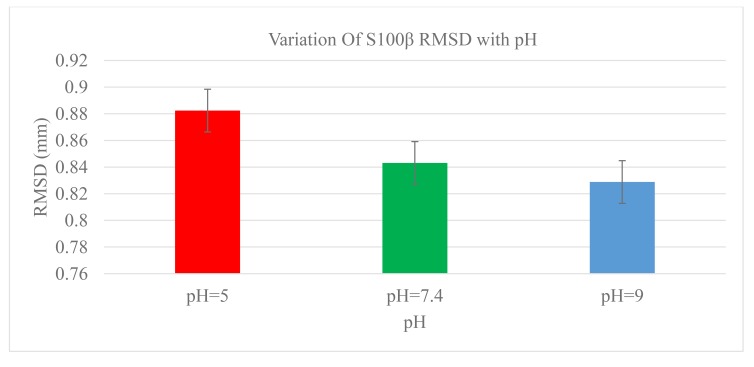
Root-mean-square deviation (RMSD) versus time graph. The graph determines the backbone fluctuations of S100β protein at varied pH values.

A statistical measure of the root-mean-square deviation (RMSD) between corresponding atoms in protein structures can aid in obtaining the protein conformational changes at different physiological parameters [36]. The structure of S100β was monitored as a function of pH and the RMSD values depicts the average distance between the backbone atoms (Fig. **2**). For the different pH 5.0, 7.4, and 9.0, considerable conformational changes were observed during the initial few picoseconds of the RMSD values (0.8824, 0.8431, and 0.8288mm), respectively. The size of the backbone fluctuation is maximum at pH~5.0 and decreases with basic pH values. Statistically, there was no significant difference in the RMSD between the physiological and basic pH values.

### 3.2 Temperature-Induced Conformational Changes

In order to understand the behaviour of S100β protein at varying temperatures (*i.e.*100 to 400K), MD simulations using the Berendsen coupling scheme in GROMACS were conducted. Graphs of RMSD, the Root-Mean-Square Fluctuation (RMSF), hydrogen bonds as well as solvent accessible surface area (SASA) versus time at the various temperatures (100K, 200K, 300K, 310K and 400K) are displayed in Fig. (**3**). As shown in Fig. (**3a**), the RMSD graph for each temperature changes significantly with 400K having the highest deviation. Simulation at 400K was basically performed to explore how higher temperatures could compromise the structural alterations of a protein. The results indicate a positive correlation between temperature and conformational stability. Further comparison of the crystallization (200K) and physiological (310K) temperatures showed a relatively low deviation in the former, depicting small deviations of the residues from the backbone as a function of time. In order to evaluate the individual residue fluctuations of the protein at the different temperatures, RMSFs were generated from trajectory analysis obtained by the molecular dynamic simulation and are shown in Fig. (**3b**). The RMSF plot at the different temperatures showed distinct fluctuations with 400K having the highest fluctuation at the protein termini.

The RMSF calculation shows that, for the entire simulation period of 1000 ps, native residues fluctuate within the range of ~0.05-0.6 nm. It can therefore be predicted that higher temperatures confer much fluctuations and flexibility in the S100β structure and that the termini are the most flexible portions of the protein. Furthermore, structural changes in the flexibility of chain A of S100β was further examined by the number of hydrogen bonds formed during the simulation experiment. Hydrogen bonds are important in predicting the fold in a protein. Variations in the number of hydrogen bonds and bond length affect the stability of a protein [37]. As indicated in Fig. (**3c**), the hydrogen bond graph showed significant variations at different temperatures (*i.e.* 100 - 400K) with 400K having the lowest number of hydrogen bonds. This could be due to the formation of more unstructured regions of S100β at higher temperatures. It can be suggested that higher temperatures result in the destruction of the hydrogen bonds holding the secondary and tertiary structures together. The SASA graph shown in Fig. (**3d**) depicts significant variations at different temperatures (*i.e.* 100K - 400K) with 400K having the highest area of approximately 76 nm^2^. This portrays that more hydrophobic residues get exposed at high temperatures which indicates denaturation marked by an increase in the exposed surface area as water solvates the unfolding proteins.

Bar graphs of RMSD, RMSF, Hydrogen bond as well as Solvent Accessible Surface Area (SASA) versus the time of the crystallization (200K) and physiological temperatures (310K) are compared and shown in Fig. (**4**). The mean RMSD for the crystallization (200K) and physiological (310K) temperatures are compared in Fig. (**4a**). The mean RMSF values for the crystallization (200K) and physiological (310K) temperatures are also compared in Fig. (**4b**). The RMSF graphs show significant variations between the crystallization and physiological temperatures. This is an indication of distinct fluctuations within the protein at the higher temperature. Also, in Fig. (**4c**), it can be observed that the individual amino acids are held by a relatively larger number of hydrogen bond networks at 200K compared to the number of hydrogen bonds at 310K. Fig. (**4d**) shows the solvent accessible surface area of the protein at 200K and 310K. At 310K, the protein becomes less compact and accordingly more regions which were initially buried at the crystallization temperature are exposed to interact with the solvent.

### 3.3 Temperature-Induced Secondary Structure Analysis

The secondary structure of a protein is the geometric structure caused by intermolecular and intramolecular hydrogen bonding of the amide groups [38]. Fig. (**7a**) shows the comparison between the secondary structure of the protein at 200K and 310K. Uniform formations of alpha helices at 200K were compared to non-uniform formation of alpha helices at 310K. The alpha helices of the protein at 200K were higher in number and more confined than that at 310K. This is due to the significant reduction and breakage of these alpha helices as a result of the destruction of the intramolecular hydrogen bonds holding the structure together. This illustrates changes in the conformational stability due to the varied metal-ion binding sites of the protein. Hence temperature appears to affect the conformational changes of S100β proteins.

### 3.4 Metal Ions Induced Conformational Changes

The conformational changes of the S100β protein was investigated at pH ~ 7.4 in the presence of Ca^2+^ since the metal ion is preferred for physiological functioning of S100β protein. The outcome of the binding was compared with other metal ions such as Zn^2+^ and Mg^2+.^ The metal ions were selected for the prediction due to the fact that they are rich in the synaptic regions of the brain where S100β protein is found. In Table **1**, unlike Zn^2+^, it is evident that Ca^2+^ and Mg^2+^ bind to the same protein residues. The residues that are in contact with Ca^2+^ and Mg^2+^include 62D, 64D, 66D, 68E and 73E, however, the binding affinity score for Ca^2+^ is higher. The RMSD was calculated for hydrogen bond interactions in the S100β protein after one least square fit for the different protein-metal ion trajectories. For the different metal ions, considerable conformational changes were observed during the early few picoseconds with RMSD value of 0.1 nm as shown in Fig. (**5a**). The RMSD graph for each of the protein-metal ion complex varied significantly with Ca^2+^-S100β complex having the highest deviation followed by Mg^2+^ and Zn^2+^. Even though, Mg^2+^ has more residue contacts compared to Zn^2+^, there is no significant deviation in terms of the RMSD between the two metal ions. Accordingly, it can be deduced that Mg^2+^ and Zn^2+^ ions confer conformational dynamics to the S100β which could compromise its cellular functions.

In order to evaluate the individual residue fluctuations of the protein-cofactor complex, RMSF graphs were generated from trajectory analysis obtained from MD simulations and the results are displayed in Fig. (**5b**). The RMSF calculations show that in the whole simulation period of 1000 ps, residues showed fluctuation values within 0.25 - 0.1 nm. Stable regions of a protein depict lesser fluctuations as compared with flexible regions. After 10 ns of simulation using GROMACS with different metal ions, it can be deduced that Zn^2+^ and Mg^2+^-S100β complexes show higher stability as compared with Ca^2+^-S100β complex. Bonds made in Zn^2+^ and Mg^2+^- S100β complexes binding sites stabilize the protein-metal ion complex and thereby reduce its flexibility depicting a unique conformational change. This flexibility appears to be accounted for by motions in the loops and hairpins around the active site of the protein.

Structural changes in the flexibility of the S100β protein were further analysed by determining the number of hydrogen bonds formed and the results are shown in Fig. (**5c**). The mean number of hydrogen bonds and distribution at varied protein-cofactor complexes were analysed. The average number of hydrogen bonds per frame is higher in Mg^2+^-S100β complex as compared with Ca^2+^ and Zn^2+^-S100β complexes. This is probably due to the high electronegativity of magnesium which favours the attraction of hydrogen atoms in the formation of hydrogen bonds. The average number of hydrogen bonds ranges from 225 to 242 at different protein-cofactor complexes. This plays relevant roles in the maintenance of the secondary and tertiary structure of a protein. Moreover, hydrogen bonds are important in predicting the specific molecular interactions in biological recognition processes of a protein. Changes in the hydrogen bond pairs within 0.35 nm distance and angle, may affect the conformational stability of a protein [37]. The hydrogen bond graph exhibited significant variations at different protein-cofactor complex, with Zn^2+^-S100β complex having the lowest number of hydrogen bonds depicting protein instability as shown in Fig. (**5c**). This is possibly due to the formation of more unstructured regions of S100β at different protein-cofactor complexes.

**Fig. (5):**
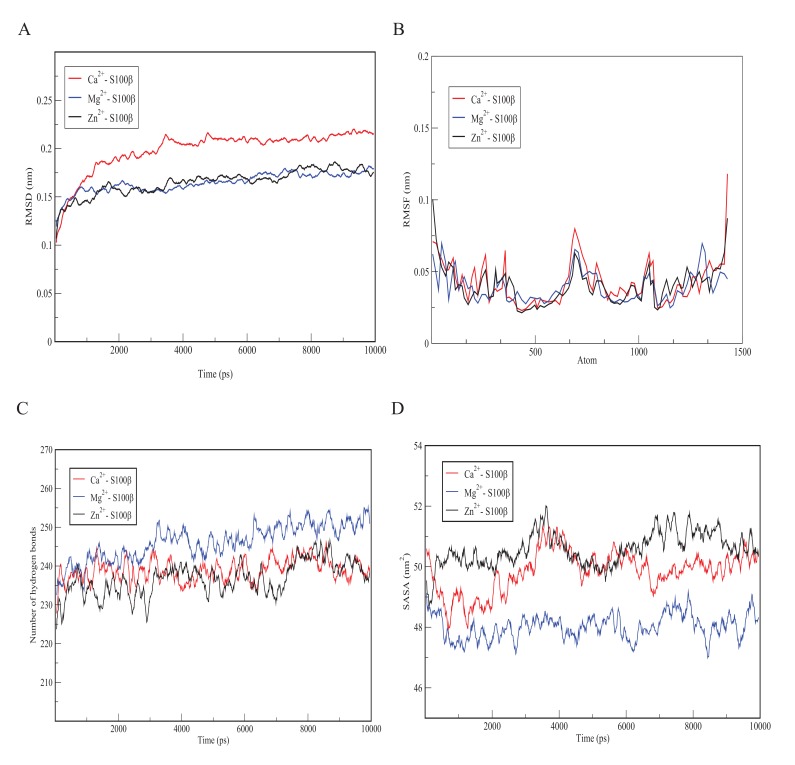
Cofactor-bound S100β analysis. **(A)** Graphs of root-mean-square deviation (RMSD); **(B)** Root- Mean-Square Fluctuation (RMSF); **(C)** hydrogen bond; and **(D)** Solvent Accessible Surface Area (SASA) for different protein-cofactors (zinc, calcium and magnesium) complexes.

The Solvent Accessible Surface Area (SASA) is a measure of the accessibility of a solvent to the hydrophobic core of a protein [39]. Variations in the solvent accessible surface area indicates the change in the exposed amino acid residues and could affect the secondary and tertiary structure of proteins. The SASA graph (Fig. **5d**) showed significant variations with Ca^2+^- S100β complex and Zn^2+^- S100β complex as having the highest exposed surface area of approximately 50.2 nm^2^. The average SASA values revealed significant difference between calcium and magnesium. This could be as a result of the burial of the hydrophobic residues into the core, signifying a conformational change in tertiary structure of the S100β protein.

From Fig. (**6a**), the binding of a cofactor to the native S100β at 310K causes a conformational change which is manifested as a disappearance of an appreciable number of hydrogen bonds. This further confirms that S100β is activated by metal-ion binding for physiological functioning. It can also be observed that Mg^2+^-bound S100β has more hydrogen bond networks present than that of Ca^2+^ and Zn^2+^-bound states.

**Fig. (6):**
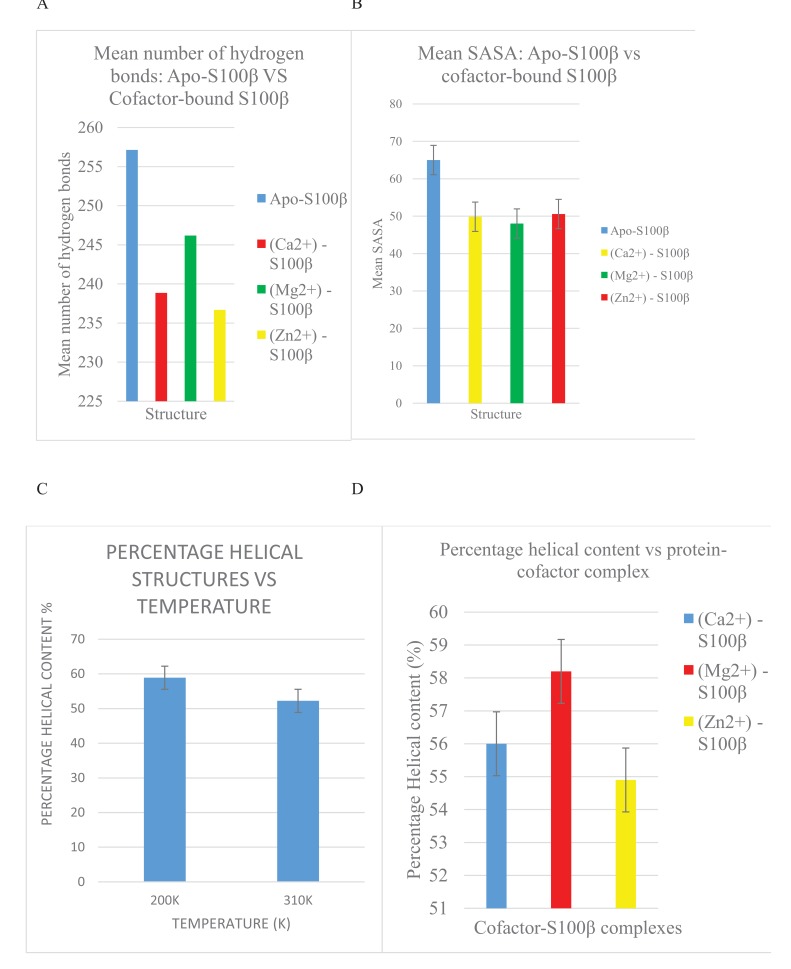
Metal ions induced conformational change studies. **(A)** Number of hydrogen bonds of apo-protein versus protein-cofactor complex; **(B)** comparison of means of Solvent Accessible Surface area of protein-cofactor complexes with apo-Protein; **(C)** secondary structure alpha- helix percentage at 200K and 310K; and **(D)** secondary structure alpha-helix percentage at varied protein-cofactor complex.

In Fig. (**6b**), the apo-S100β has more residues interacting with the solvent than the cofactor- bound S100β protein at 37°C (310K). The cofactor-bound protein has more regions buried in the hydrophobic core and hence less surface area accessible to the solvent. This shift in the unfolding of buried residues from the hydrophobic core signifies conformational alterations triggered by cofactors at the physiological temperature. Moreover, Fig. (**6c**) shows the changes in the secondary structure of the alpha-helices as a function of temperature. From the same bar graph, the 310K had lower percentage of alpha helical structures as compared to 200K. As helical content of a protein determines its stability, the more the alpha-helix present in a protein structure, the more stable it is [4]. Accordingly, the protein misfolds as temperature increases thereby causing a conformational change in the S100β protein. Also, Fig. (**6d**) indicates the changes in the secondary structure alpha-helices with different cofactors of the protein. From the bar graph (Fig. **6d**), Zn^2+^-S100β also had the lowest percentage of helical content. Hence, the different cofactors-bound S100β protein affects the conformational changes of S100β proteins.

### 3.5 Secondary Structure Analysis of Protein-Cofactor Complexes

Fig. (**7b**) shows the number of residues and the corresponding secondary structures in each frame of the MD simulations for different cofactors bound to S100β. The secondary structure analyses were conducted to elucidate the regions that are essential in the structural changes of a protein caused by intermolecular and intramolecular hydrogen bonding of the amide group [22]. Highly fluctuated residues show a higher inclination for the formation of unstructured protein regions than regular α-helical and β-strands secondary structural elements as depicted in the RMSF graph at different protein-cofactor complexes. Additionally, it shows the comparison between the secondary structures of the respective protein-cofactor complexes. Uniform formation of alpha helices at residue 3 to 16 for Ca^2+^-bound protein was identified and compared to a non-uniform formation of alpha helices at residue 15 to 19 for Zn^2+^-bound S100β protein secondary structure. The alpha helices of the Mg^2+^-S100β was higher in number and confined relative to Zn^2+^-S100β with the characteristic appearance of two beta hairpins.

## 4 SUMMARY OF FINDINGS AND ITS IMPLICATIONS

As part of the model verification, the different binding domains of the cofactors identified were closely related to experimental binding positions of typical EF-hand (EF-2: D61-E72) [12]. The molecular dynamics simulations of the different protein-cofactor complex appear to have minor structural changes when Ca^2+^ is bound to the pseudo-EF-hand as opposed to the typical EF-hand. Different S100β structural alterations which could be biologically relevant were identified as a result of the weak binding affinity of the cofactors. The low binding affinity of the cofactors appear to trigger the higher concentrations of S100β proteins in cells without depleting the cofactors levels [40]. The molecular dynamics simulations also corroborate that Ca^2+^, Mg^2+^ and Zn^2+^ S100β complexes are relevant secondary messengers in all living cells, since these cofactors helps to maintain the integrity of ion signalling and transmission in a spatially coordinated manner.

Moreover, the different binding domains of the cofactors divulges the varied affinities of the divalent metal ions. This may also help in understanding oligomerization properties as well as posttranslational modification enhancement (relevant in drug resistance) in the characterization of S100β proteins. Also, Ca^2+^-bound S100β proteins appears not to only cause structural transitions and alterations but also results in a tighter dissemination of helices within the EF-2 binding motif relevant for target protein interactions. Additionally, acidic pH (~5.0) appears to confer conformational instability to S100β proteins. Protein misfolds at higher temperatures by the disappearance of helical regions which are responsible for proper folding. It was observed that at 100K and 400K, alpha helical content was 57.8% and 43.3%, respectively.

## CONCLUSION

The study aimed to investigate the impact of physicochemical factors such as temperature, pH and selected cofactors on the conformation dynamics of S100β protein. There were significant changes that were observed in the conformation and folding patterns of S100β protein and how they relate to the type of bound co-factor (Ca^2+^, Mg^2+^ and Zn^2+^). The backbone fluctuations of the S100β protein were shown to be higher in acidic medium. The two binding motifs of the protein were not affected appreciably by varying temperatures. The studies provide great potential for experimental and structural characterisation of the S100β protein. Even though, these findings are primarily based on computational predictions; experimental validation could enrich existing efforts geared towards exploiting S100β as a potent diagnostic biomarker as well as the design of novel drugs.

## Figures and Tables

**Fig. (3):**
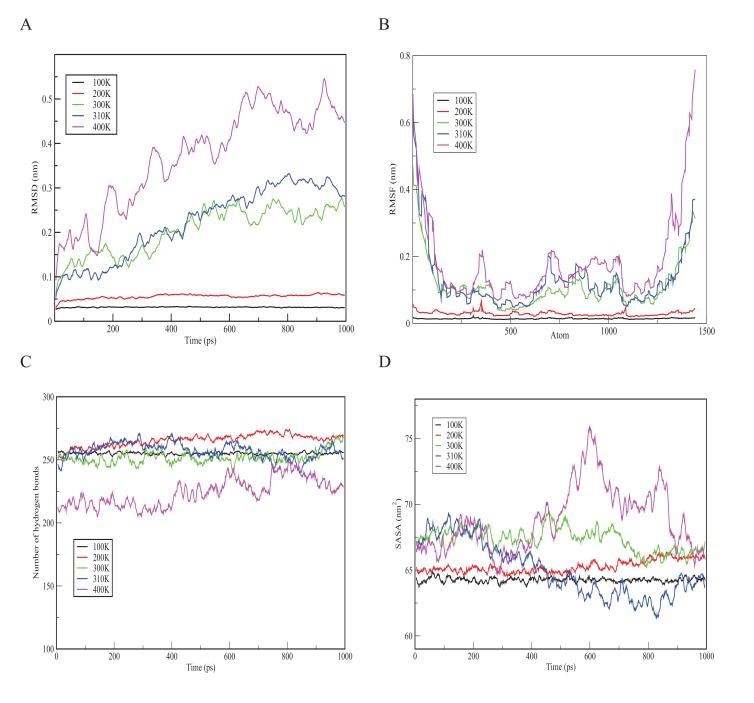
Temperature variations analysis. **(A)** Root-mean-square deviation (RMSD); **(B)** root-mean- square fluctuation (RMSF); **(C)** hydrogen bond; and **(D)** Solvent Accessible Surface Area (SASA) versus time at varied temperatures.

**Fig. (4):**
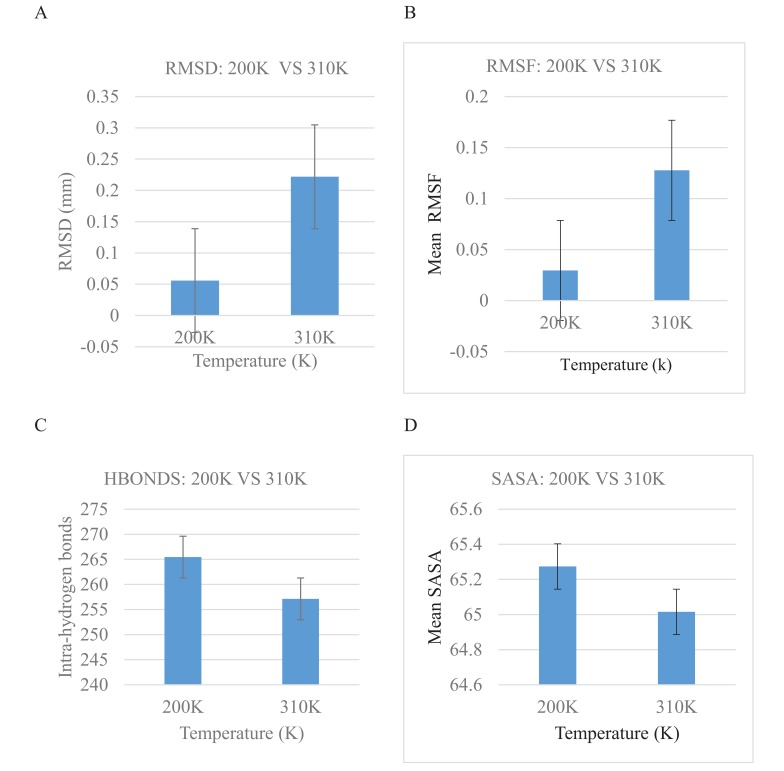
Physiological and crystallization temperature analysis. **(A)** Root-mean-square deviation (RMSD); **(B)** root-mean-square fluctuation (RMSF); **(C)** hydrogen bond; and **(D)** Solvent Accessible Surface Area (SASA) of S100β protein bar graphs compared at 200K and 310K.

**Fig. (7):**
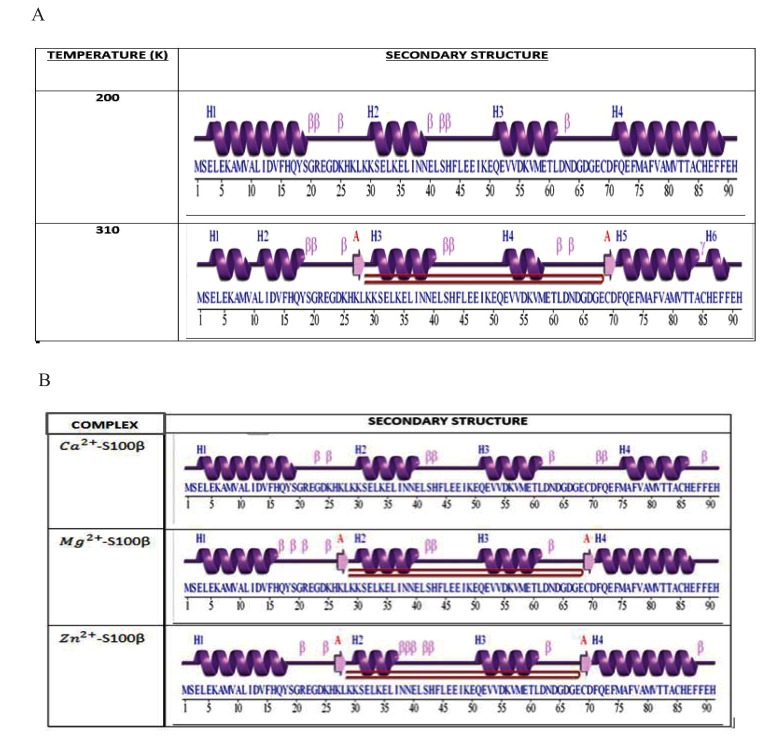
Secondary structure analysis. **(A)** Changes in the secondary structure at 200K and 310K; and **(B)** changes in the secondary structure at varied protein-cofactor complexes.

**Table 1:** Metal Ion Binding (MIB) Server [33] results showing the different binding affinities and the different interacting residues per cofactor-bound S100β protein with the cofactors represented as spherical balls.

Cofactor-Bond S100β	Binding Residues	Binding Scores	3D Model of the Best Binding Mode
Ca ^2+^ - S100β	62D, 64D, 66D, 68E, 73E	2.891	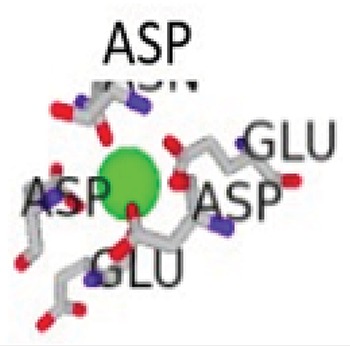
	62D, 64D, 65G, 66D, 68E, 69C, 73E	2.766	
Mg^2+^ - S100β	62D, 64D, 66D, 68E	2.031	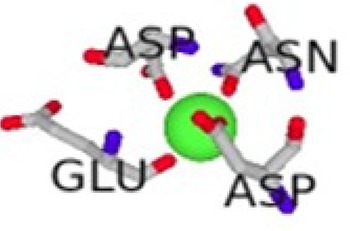
	62D, 64D, 66D, 68E, 73E	1.944	
Zn^2+^ - S100β	86H, 87E	1.538	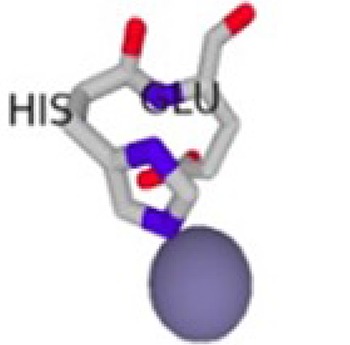
	55D, 59E	1.480	
